# Thigh Abscess as an Uncommon Complication of Left-Sided Colonic Diverticulitis and the Pitfalls in Treatment: An Interesting Case Report

**DOI:** 10.7759/cureus.23927

**Published:** 2022-04-07

**Authors:** Aditya Soni, Sandeep Munshi, Kapil Shirodkar, Ashutosh Soni, Ajay Dhanopeya, Niranj G Radhamony, Sachith Sreenivasan

**Affiliations:** 1 Trauma and Orthopaedics, Furness General Hospital, Barrow-in-Furness, GBR; 2 Radiology, Royal Lancaster Infirmary, Lancaster, GBR; 3 Minimally Invasive and General Surgery, Bombay Hospital, Indore, IND; 4 Trauma and Orthopaedics, Chandra Kant Birla Hospital, New Delhi, IND; 5 Trauma and Orthopaedics, Royal Stoke University Hospital, Stoke-on-Trent, GBR; 6 General Surgery, Auckland City Hospital, Auckland, NZL

**Keywords:** diverticulitis connection to thigh, intra-abdominal connection to thigh, incision and drainage, hartmann’s procedure, thigh abscess, sigmoid diverticulitis

## Abstract

Colonic diverticulitis is one of the common causes of surgical intervention in general surgical practice. In most cases, surgical intervention depends on the presence of a collection around the sigmoid colon, the feasibility of percutaneous drainage, and the patient’s medical condition. The collection can occur in the thigh in rare cases due to a fistulous communication with the retroperitoneum and can track gravitationally along the psoas muscle into the thigh and leg without any discernible collection around the sigmoid colon or in the abdominal cavity. We came across a similar case of a 54-year-old man who presented with abdominal pain, and thigh and leg swelling. Left-sided colonic diverticulitis was seen without any discernible abdominal collection and a thigh abscess during the initial clinical presentation. He was treated with multiple drainages of the thigh abscess, ultimately followed by a Hartmann’s procedure over a total hospital admission period of 52 days. Current literature does not throw much light on such a situation and makes it all the more critical to illustrate this case. We present this rare case and give a complete account of investigations, disease course, and the interventions done to throw light on the optimal management of such cases.

## Introduction

Colonic diverticulitis is one of the dreaded differentials for left iliac fossa pain and is known to be caused due to low intake of fibers in the diet and an increase in the intraluminal pressure due to motility abnormalities [1]. Left-sided colonic diverticulitis usually presents with typical signs of pain in the left iliac fossa with systemic symptoms, while the grave complications include perforation, colovesicle, and colovaginal fistulas [2]. On the other hand, perforated diverticulitis is even worse with overt abdominal signs of fecal peritonitis and septic shock. Diverticulitis has been traditionally managed conservatively if no collection is found in the abdomen, while a two-stage intervention including percutaneous aspiration followed by resection of the affected segment is advocated in severe cases of perforation and intrabdominal collection on a computed tomography (CT) scan [3]. 

However, in rare instances, the complications of colonic diverticulitis can be devastating. We present a rare case of sigmoid diverticulitis without any abdominal paracolic collection, presenting with a fulminant abscess in the thigh and masquerading with musculoskeletal complaints rather than abdominal symptomatology. Only a few prior documented case reports in the published literature are available for review [4,5,6], and it highlights the need for swift abdominal intervention in such cases, irrespective of the abdominal collection.

## Case presentation

A 54-year-old male was admitted under the care of an acute medical team with a three-day history of abdominal pain, loose stool, and vomiting. Additionally, he had a two-week history of gradually increasing left thigh swelling originating in the inguinal region and spreading distally. On examination, he had tenderness over his left iliac fossa and a tense left thigh swelling along the posteromedial aspect of his left distal thigh extending up to the medial aspect of the knee joint. Since he was febrile but not in shock, along with erythema and swelling in the thigh and leg, the medical team formulated provisional clinical diagnoses of gastroenteritis along with cellulitis and possible deep vein thrombosis (DVT). Relevant laboratory work-up revealed raised C-reactive protein (CRP) (332.5 mg/L), high white cell counts (25.8 x 10^3^/mL ) with predominant neutrophilia (22 x 109/L), low estimated glomerular filtration rate (28 ml/min/1.73 m2), and mildly elevated D-dimer levels. His co-morbidities included a high BMI of 45 and bronchial asthma. 

**Figure 1:**
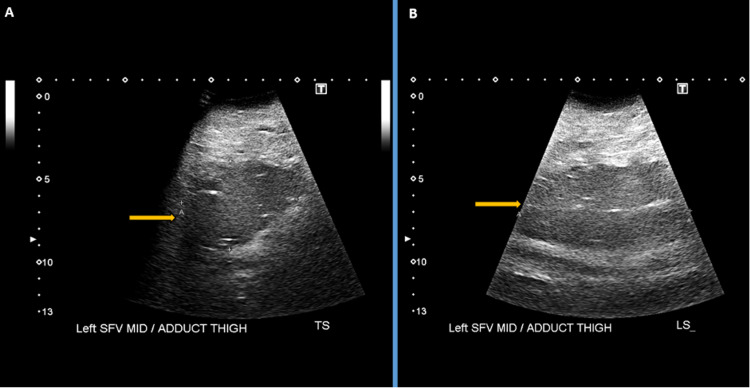
Pre-operative greyscale ultrasound transverse (A) and longitudinal (B) images showing a deep-seated intramuscular collection in the left mid-thigh represented by orange arrows

On venous Doppler imaging on day one, DVT was excluded; however, there was incidental detection of a deep-seated intramuscular fluid collection with dense internal echoes within the deep tissue of the left mid-thigh and was thought to represent either an abscess or a hematoma likely (Figure 1). Following this, the orthopaedic and surgical teams were alerted and got involved in the care.

**Figure 2:**
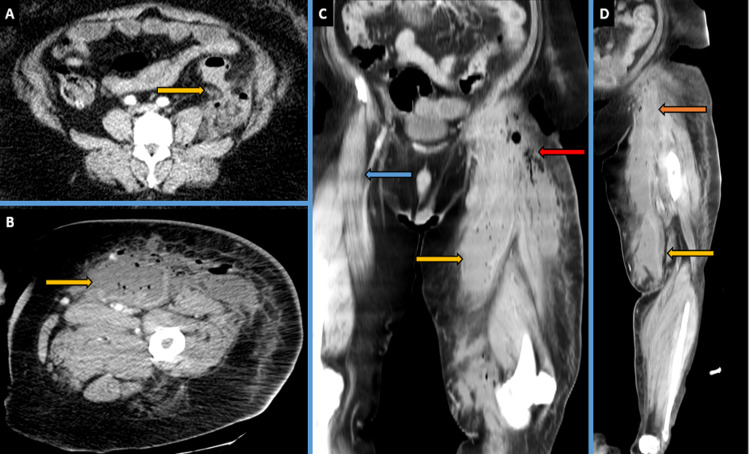
Pre-operative day one CT images (A) Axial contrast-enhanced computerised tomography (CECT) image showing left-sided colonic diverticulitis with surrounding inflammatory changes. (B) Axial CECT image of the left upper thigh showing rim enhancing abscess. (C) Coronal CECT image showing the supero-inferior extent of the left thigh abscess extending from the left inguinal region up to the left of the knee by yellow/red arrows, blue arrow depicting the normal side. (D) Oblique coronal CECT image showing the supero-inferior extent of the left thigh abscess extending from the left inguinal region up to the knee level.

On the advice of the surgical team, a subsequent CT angiogram of the lower limb on the same day was performed to ascertain the further nature of this collection, which revealed left-sided colonic diverticulitis without any well-formed intra-abdominal collection. An additional rim-enhancing abscess was also seen in the left thigh tracking from the left psoas muscle intra-abdominally up to its distal attachment on the medial femur and tracking down further inferiorly and reaching the knee level as seen in Figure 2.

After a multidisciplinary team meeting (MDT) involving general surgery and orthopaedics, a CT-guided intra-abdominal drainage was requested the same day, along with a decision to drain the thigh abscess on an urgent basis. Radiologists deferred the percutaneous drainage due to the lack of well-formed drainable retroperitoneal collection and further deterioration of the patient’s clinical condition. The thigh abscess drainage was carried out as a priority by the orthopaedic team, along with conservative non-surgical management for his diverticulitis with appropriate antibiotic coverage as decided by the general surgical team. On incising the most dependent part of the thigh abscess at the posteromedial aspect of the distal thigh, there was a gush of foul-smelling pus (2 liters), which was evacuated, and a surgical drain bag was attached to the surgical thigh wound, which was kept open. The patient continued to have abdominal symptoms, and a re-look thigh washout was done two days later. Following the first surgery, there was a continuous drain of up to 100ml pus per day from the incision, which continued even after the second washout. The culture sensitivity on the pus from the thigh wound showed anaerobic infection and isolated bacteria *Escherichia coli* (*E. coli*), hinting toward a bowel-related source of the infection. The patient’s inflammatory parameters started coming down, he was no longer septic, but he did not improve in terms of abdominal pain and continued having intermittent fever episodes. The antibiotic cover over the whole course of treatment included intravenous (IV) co-amoxiclav and clindamycin initially, later followed by a change to IV piperacillin with tazobactam.

**Figure 3:**
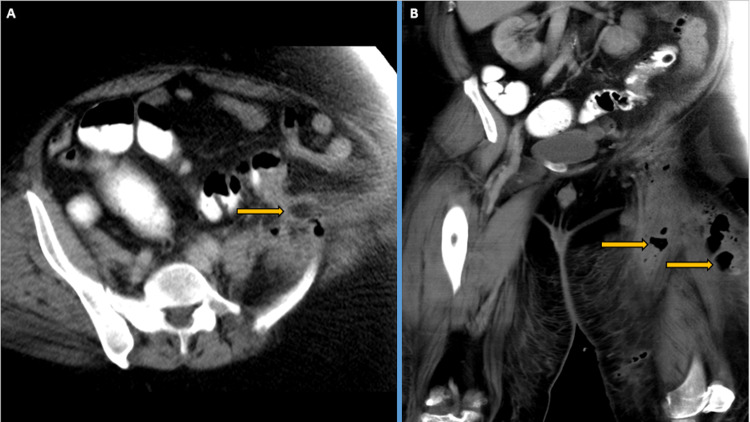
Day eight CT (A) Axial contrast-enhanced computerised tomography (CECT) pelvis image showing persistence of the fistulous tract from the left-sided descending colon extending to the left-sided retroperitoneum overlying the left iliopsoas. (B) Coronal CECT image showing interval regression of the liquified thigh abscess with residual few air pockets in the left proximal thigh, marked with arrows.

A second CT, which was a contrast-enhanced CT (CECT) to check the progress of the diverticulitis, was done on day eight, which showed an interval regression of the thigh abscess, and a few residual air pockets in the thigh abscess with persistent diverticular inflammatory changes in the left iliac fossa. There was a possible fistulous communicating tract extending from the persistent peri-colonic inflammatory changes to the retroperitoneal left psoas muscle, as seen in Figure 3. The patient was still being treated with antibiotics for the diverticulitis during this time. 

**Figure 4:**
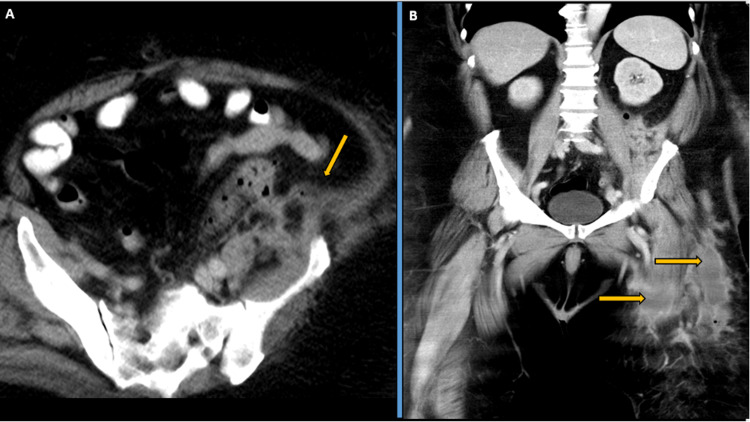
Day 18 CT (A) Axial contrast-enhanced computerised tomography (CECT) pelvis image: yellow arrows showing persistence of the fistulous tract from the left-sided descending colon extending to the left-sided retroperitoneum overlying the left iliopsoas. (B) Coronal CECT image with yellow arrows showing interval re-formation of the left thigh abscess with a liquified component.

**Figure 5:**
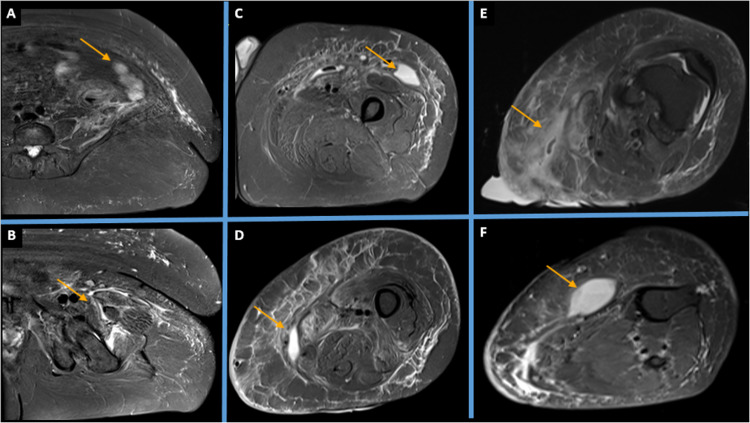
Day 22 MRI Axial short tau inversion recovery (STIR) images at the level of (A) pelvis, (B) proximal left thigh, (C) mid left thigh, (D) lower left thigh, (E) left knee joint, and (F) proximal left leg, with orange arrows showing infective fluid pockets.

Since the patient did not improve in terms of abdominal pain, and there was a repeat rise in inflammatory markers (CRP 270 mg/L, white cell counts 19 x 10^3^/mL) in the days after the second surgery, a third contrast-enhanced CT was done on day 18. It showed re-progression of the thigh abscess with the interval appearance of a liquefied thigh abscess, as seen in Figure 4. It was further confirmed on a subsequent MRI of the pelvis and thigh performed on day 22, which showed a persistent left thigh abscess with surrounding mild infective myositis and non-resolving diverticulitis with a possible fistulous tract between the sigmoid diverticulum and the retroperitoneum as seen in Figure 5. As there was no interval intra-abdominal collection, the general surgery team continued with conservative antibiotic management.

The orthopaedic team performed a third thigh washout on day 25 due to non-ceasing pus drainage from the thigh incision and interval resurgence of thigh swelling due to re-accumulation of pus within the thigh. Unfortunately, this drainage also did not yield any improvement in terms of abdominal pain. There was a marginal drop in inflammatory markers with CRP (245 mg/L) and white cell counts (18 x 10^3^/mL), but they were still very high, and the patient’s hospital stay was extended.

**Figure 6:**
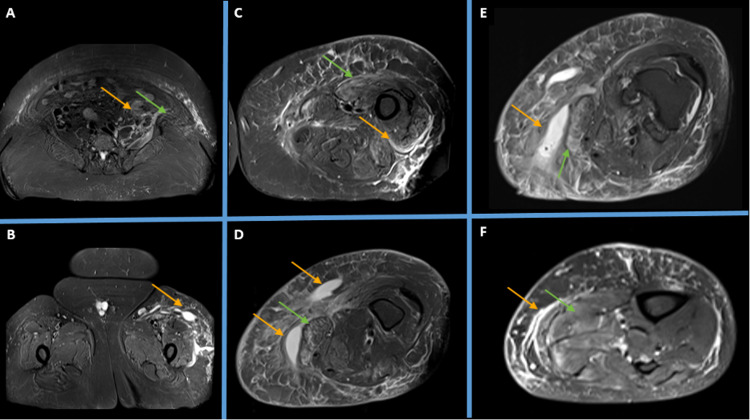
Day 36 MRI Axial short tau inversion recovery (STIR) images at the level of (A) pelvis, (B) proximal left thigh, (C) mid left thigh, (D) lower left thigh, (E) left knee joint, and (F) proximal left leg, with orange arrows showing persistent infective fluid pockets and green arrows showing interval infective myositis.

Repeat MRI imaging on day 36 showed the presence of a few smaller recurrent collections in the medial thigh with infective myositis and again showed persistent inflammatory changes in the left iliac fossa without any demonstrable well-formed intra-abdominal collection, as seen in Figure 6. Conservative management for the diverticulitis was continued.

**Figure 7:**
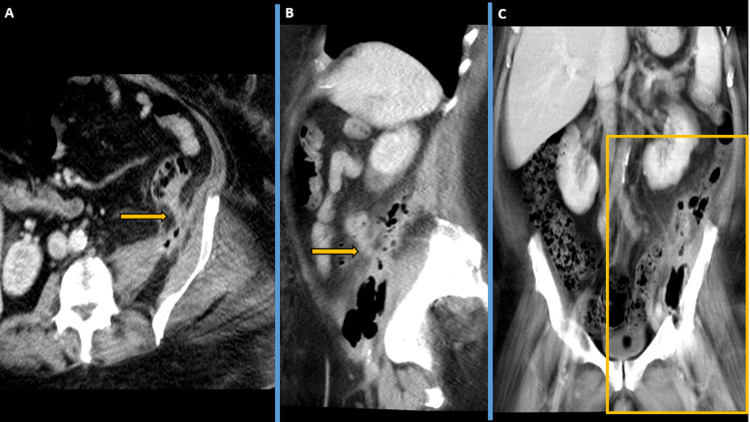
Day 42 CECT: (A) Axial contrast-enhanced computerised tomography (CECT) pelvis image shows persistent fistulous tract from the left-sided descending colon extending to the left-sided retroperitoneum overlying the left iliopsoas. (B) Sagittal oblique CECT image showing persistent fistulous tract from the left-sided descending colon extending to the left-sided retroperitoneum overlying the left iliopsoas. (C) Coronal CECT image showing left-sided retroperitoneal collection/abscess involving the left iliopsoas group of muscles, shown further in Figure 8.

**Figure 8:**
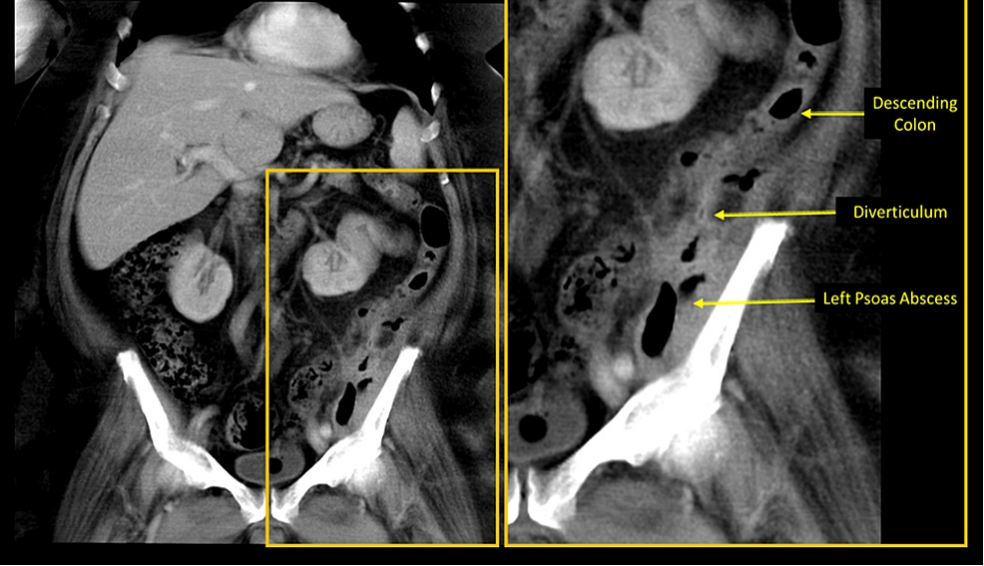
Day 42 CECT Coronal section showing persistent fistulous tract from the left-sided descending colon extending to the left-sided retroperitoneum overlying the left iliopsoas. CECT- contrast-enhanced computerised tomography

Due to the patient being no better and the reluctance of the general surgical team to intervene, a fourth thigh washout was done by the orthopaedic team on day 40 with further incisions on the anterolateral aspect of the thigh, coinciding with the collections seen there in the previous MRI with only small pus drainage from these incisions at this setting. The patient started deteriorating and became septic the next day with persistent fever and increasing abdominal pain; hence a fourth contrast-enhanced CT on day 42 for the first time showed an intra-abdominal retroperitoneal collection around the left-sided colon measuring 15 x 6.5 x 3.5cm as seen in Figure 7, 8.

This was followed by an urgent Hartmann’s procedure on day 42, following which the patient improved, and no further collections in the thigh were identified. Hartmann’s procedure was technically difficult due to the morbid obesity of the patient, and it revealed an inflamed, thickened segment of the sigmoid colon adherent to the left pelvic brim and left lateral and posterior abdominal wall which required meticulous dissection. There was a perforation identified intraoperatively, but the whole procedure was completed with no untoward incidents. The patient improved remarkably over the next couple of days without any complications and required no further thigh washouts. He was ultimately discharged in a non-febrile state a week later with appropriate antibiotic coverage. The patient’s condition continued to improve over the next couple of weeks, and he was followed up for two months by both the orthopaedic and general surgery team and finally discharged from our care without requiring any further follow-ups. The Hartmann’s procedure done was not reversed by the surgical team on follow-ups due to the risk of complications. Additionally, the posteromedial distal thigh wound was kept open throughout the entire time; it was followed up by the community nurse for a couple of months with regular dressings until it healed with secondary intention.

Just before Hartmann’s procedure, during the same anaesthetic, cystoscopy and ureteric stenting on the left side were performed on the table as a prophylactic measure which was removed after the abdominal procedure. The stenting was done to mitigate any problems should the ureter get damaged during the complex surgical dissections required in the procedure. 

## Discussion

Perforation of sigmoid diverticulitis lands it directly into the complicated category, which is known to have some kind of morbidity associated with it after surgery in 43% of the cases [7] while having a mortality of around 10% for perforated cases treated promptly [8] and 31% is seen at six months for fecal peritonitis cases [9]. Among the complications of complicated diverticulitis are bleeding, a fistula formation, intra-abdominal abscess, perforation, phlegmon, stricture causing colonic obstruction, of which the most common is phlegmon and abscess at 31% each with fistula the least common at around 5% [10]. It is also seen that 25% of these patients, after surgery, land up in an ICU, and the total hospital stay in complicated diverticulitis managed adequately has been around 17 to 19 days [7,10].

The case report highlights the problems of delayed abdominal intervention in complicated diverticulitis cases due to the paucity of literature on such cases and hence the lack of awareness about it. Thigh collections originating from other areas such as psoas abscess [11], post malignancy colorectal surgeries [12], and renal tuberculosis have been reported previously in the literature [13]. There are only a few published cases of an intrabdominal retroperitoneal infection and neoplasms causing a thigh abscess secondary to a communicating tract [14]. 

The paucity of literature makes managing such cases difficult, with cases often having delayed recovery due to late gastrointestinal surgical intervention in the absence of a well-formed intra-abdominal/retroperitoneal collection. Often such cases are managed conservatively due to the reluctance of the GI surgical team to intervene in the absence of an intraabdominal collection. A good clinical examination followed by a CT scan of the pelvis and abdomen, which is the gold standard investigation, should be followed in each case with such presentations. It has been seen that, along with clinical acumen, multidisciplinary meetings go a long way in improving outcomes [15-17].

In our case, the patient was primarily managed by the orthopaedic department due to the initial presentation of a thigh abscess and the absence of intra-abdominal collection. The immediate thigh abscess drainage as an emergency life-saving measure was warranted in this acutely septic patient; the thigh abscess was possibly recurring due to gravity dependant tracking of the infective fluid along the left-sided retroperitoneum through the communicating fistulous tract into the left proximal thigh and extending into the proximal leg. The patient underwent repeated surgeries to drain recurring thigh abscesses when perhaps post-inflammatory diverticular perforation and secondary fistulisation were possibly the primary source of infection and could have been tackled with an earlier GI surgical intervention. The Hartmann’s procedure eventually done is the gold standard treatment in acute septic diverticular perforation cases [18].

The dilemma now comes about the best timing of abdominal intervention, which in this case, we believe was possible after the second washout of the thigh, when the pus continued to drain. An MDT between the general and orthopaedic surgeons after incomplete response after each washout regarding further surgical intra-abdominal management could have helped in faster recovery. On reflection, the delayed intra-abdominal surgical intervention resulted in a prolonged hospital stay of 52 days and four thigh washouts along with the abdominal surgery, not excluding the patient’s surgical morbidity, increased interval radiation exposure, and additional financial costs to the hospital. Additionally, the patient caught a COVID-19 infection during the prolonged hospital stay but luckily fought through it and recovered uneventfully.

## Conclusions

Cases with diverticular perforation with uncommon complications need a multidisciplinary holistic approach to improve patients’ recovery and reduce hospital stay and morbidity. A paucity of published literature regarding the atypical and uncommon complications of colonic diverticular perforation itself can complicate matters. As seen, thigh abscesses can masquerade as the initial clinical presentation in left-sided colonic diverticular perforations instead of an intra-abdominal collection, in which case, along with the help of CT, we believe a detailed clinical examination in the first instance followed by continued interdepartmental discussion/communication is the best way forward to decide timely and effective interventions. Additionally, in such non-responding cases after surgical thigh drainage who have a background of perforated diverticulitis, a communicating tract should be suspected between the abdomen and the thigh. Once evidenced on the CT/MRI, Hartmann’s procedure should be offered earlier after careful consideration, even without a demonstrable intra-abdominal collection.

## References

[1] Painter NS, Truelove SC, Ardran GM, Tuckey M (1968). Segmentation and the localization of intraluminal pressure in the human colon, with special reference to the pathogenesis of colonic diverticula. Gastroenterology.

[2] Floch MH, Bina I (2004). The natural history of diverticulitis: fact and theory. J Clin Gastroenterol.

[3] Wong WD, Wexner SD, Lowry A (2000). Practice parameters for the treatment of sigmoid diverticulitis - supporting documentation. Dis Colon Rectum.

[4] Murphy PB, Belliveau P (2012). Left-sided sigmoid diverticulitis presenting as right-sided thigh abscess. Int Surg.

[5] Wadood A, Odeh A, Rana K, Zaman S (2017). A rare case of sigmoid colon perforation with subsequent psoas abscess collection with extensive involvement of the sartorius muscle. BMJ Case Rep.

[6] Peacock JE Jr (1982). Colonic perforation with thigh abscess: an unusual presentation of tuberculous spondylitis. South Med J.

[7] Schwesinger WH, Page CP, Gaskill HV 3rd, Steward RM, Chopra S, Strodel WE, Sirinek KR (2000). Operative management of diverticular emergencies: strategies and outcomes. Arch Surg.

[8] Sartelli M, Weber DG, Kluger Y (2020). 2020 update of the WSES guidelines for the management of acute colonic diverticulitis in the emergency setting. World J Emerg Surg.

[9] Eckmann C, Bassetti M (2014). Prognostic factors for mortality in (fecal) peritonitis: back to the roots!. Intensive Care Med.

[10] Hussain A, Mahmood H, Subhas G, El-Hasani S (2008). Complicated diverticular disease of the colon, do we need to change the classical approach, a retrospective study of 110 patients in southeast England. World J Emerg Surg.

[11] Petrovic I, Pecin I, Prutki M (2015). Thigh abscess as an extension of psoas abscess: the first manifestation of perforated appendiceal adenocarcinoma: case report. Wien Klin Wochenschr.

[12] Simsek A (2021). Thigh abscess secondary to intra-abdominal pathologic conditions: three cases progressing to necrotizing fasciitis. Wounds.

[13] Dharmapalan A, Vijaykumar R, Bhoopal S (2013). Renal tuberculosis presenting as thigh abscess. Indian J Surg.

[14] Rotstein OD, Pruett TL, Simmons RL (1986). Thigh abscess. An uncommon presentation of intraabdominal sepsis. Am J Surg.

[15] Sartelli M, Chichom-Mefire A, Labricciosa FM (2017). The management of intra-abdominal infections from a global perspective: 2017 WSES guidelines for management of intra-abdominal infections. World J Emerg Surg.

[16] Lee KH, Sohn MK, Jeong HS (2018). The effect of inter-departmental stroke meetings on rehabilitation in a comprehensive cerebrovascular center. J Korean Med Sci.

[17] Amin KH (2015). Multidisciplinary team meeting in UK geriatric medicine. Journal of gerontology and geriatric research.

[18] Lumpkin ST, Chaumont N (2019). Management of freely perforated diverticulitis. Dis Colon Rectum.

